# Getting to First Base: Developmental Trajectories of Major League Baseball Players

**DOI:** 10.3389/fpsyg.2019.02563

**Published:** 2019-11-14

**Authors:** Matthew McCue, Joseph Baker, Srdjan Lemez, Nick Wattie

**Affiliations:** ^1^Faculty of Health Sciences, Ontario Tech University, Oshawa, ON, Canada; ^2^School of Kinesiology and Health Sciences, York University, Toronto, ON, Canada; ^3^Kinesiology and Health Promotion Department, California State Polytechnic University, Pomona, CA, United States

**Keywords:** pathways, high performance, sport, talent identification, athlete development

## Abstract

The road to professional baseball illustrates the complexity, variability and non-linearity of athlete development, as players may be drafted from various high school, 2 year, or 4 year programs and then placed into extensive minor league systems. The purpose of this study was to identify the different pathways to Major League Baseball (MLB) and explore their influence on career success. Performance and developmental data of 2,291 American-born MLB players who debuted between 1990 and 2010 were collected using baseball-reference.com. Three performance indicators, career games played (GP), age of debut, and wins above replacement (WAR; player’s total contributions in wins), were coupled with high school, post-secondary, and Minor League Baseball (MiLB) data. Analyses revealed 17 descriptively different pathways to MLB, which were grouped into three main streams based on the last institution attended before entrance into professional baseball. Overall, 63% of the athletes started their career directly after attending a 4 year higher education institution, 23% after high school, and 14% directly after attending a 2 year institution. Interestingly, 78% of the athletes did not sign or were not selected as high school draft picks. Position players drafted or signed from high school debuted in MLB younger (*M* = 23.99) and averaged significantly more MiLB GP (*M* = 909.13) than those drafted or signed from a 2 year (*M* = 25.67 and 834.41 GP) or 4 year institution (*M* = 25.95 and 752.33 GP). Pitchers signed or drafted from high school also debuted in MLB younger and played more MiLB games, as well as played in more MLB games than players from a 2 year or 4 year institution, *F*(8, 3,082) = 31.96, *p* ≤ 0.001. No significant differences of WAR were noted in position players or pitchers. Perhaps pitchers who are drafted from high school are afforded more opportunities to succeed, which may be indicative of sunk cost effects. This is conceivable as these players had the highest average GP but did not accrue a higher WAR. Future research may benefit from the consideration of post-secondary and/or high school statistics in combination with draft selection data, which may have important implications for improving talent identification accuracy.

## Introduction

The sporting entry draft and the ability (or lack thereof) of professional front offices to successfully identify high-caliber players is well researched (Simpson, 1990; Staw and Hoang, 1995; Spurr, 2000; Popper, 2004; Koz et al., 2012). One of the challenges of navigating elite sport entry drafts is that athletes may enter the draft having experienced different athlete development contexts and may have taken different pathways within those contexts. This complicates decisions and judgments about talent and potential, and may be a constraint for Major League Baseball (MLB) in particular. Analyses of MLB’s first-year player draft often yields results that are significantly different from the three other major North American sports (National Football League – NFL, National Basketball Association – NBA, National Hockey League – NHL). Koz et al. (2012) demonstrated this when the relationship between career games played (GP) and draft round were explored across MLB, NBA, NHL, and NFL. Unlike the three other sports, no significant differences were found between draft rounds and GP in MLB. Of course, the ideal drafting strategy would be to use early round draft picks to select players with the best potential for performance (career GP) as the cost for these selections (in terms of contracts and bonuses), compared to late round selections, is much higher (Massey and Thaler, 2013). However, this strategy appears to be extremely difficult to implement in the MLB market. In fact, a study of first round draftees selected between 1965 and 1985 found that only 63% of those players played even one game at the major league level (Spurr, 2000).

An obstacle that MLB front offices face is consolidating the abundance of information on developing baseball players. The explosion of “sabermetrics,” a term coined by Bill James to explain the analysis of performance data via detailed statistics (see Beneventano et al., 2012; Costa et al., 2014, for extensive reviews and background information) has revolutionized how professional clubs interpret the efficiency of players. However, the lack of verifiability in performance statistics from the different player development pathways (e.g., collegiate vs. high school levels) largely restricts sabermetrics to analyzing players already in MLB. Moreover, the quality of different athlete development contexts and pathways may make it difficult to appraise an athlete’s skill level and potential. As such, there is a need for research that explores the influence of variation in pathways to elite sport.

Research dedicated to describing optimal sport participation and athlete development pathways typically focuses on youth sport and appropriate *types* of participation. These trajectories and stages typically focus on the optimal and sub-optimal (according to their proponents) qualitative and quantitative characteristics of sport participation. In a model like the Developmental Model of Sport Participation (DMSP; Côté et al., 2007; Fraser-Thomas and Côté, 2009), these characteristics include whether athletes participate in one sport or multiple, the focus on play and intrinsic motivation, the focus on effortful practice (deliberate practice), and at what ages these activities occur. Similarly, the Foundation, Talent, Elite, Master (FTEM) model describes a number of stages (see Gulbin J.P. et al., 2013; Weissensteiner, 2015): foundational (i.e., learning and refining basic movement skills, and eventual commitment to sport), talent (i.e., demonstration and verification of talent, and initial success and reward for performance), elite (achievement of status and success at elite/professional levels), and mastery (success over multiple years). Like the DMSP, each stage is also associated with “best practices” or ideal characteristics (Weissensteiner, 2015). In some ways, such models attempt to summarize higher order themes and stages in order to be as generalizable as possible.

One of the challenges to such models is accounting for the fact that there can be notable variation in the pathways and opportunities *within* the stages described in many athlete development models. Whether it is the “pre-elite” phase of the FTEM, the “training to compete stage” of long-term athlete development (LTAD), or the “investment years” stage of the DMSP, these models and phases do not account for the significant variation in the contexts that athletes may be participating in. For example, in each of the aforementioned stages, an athlete could be participating in an elite amateur developmental league, in university/college sport, or within club system. Indeed, it is not clear how variations in *the contexts in which* athletes participate during certain stages of athlete development influence the likelihood of future success in elite sport.

The road to MLB, with the various collegiate programs across North America and its extensive minor league system, is an excellent illustration of the potential importance different pathways have on athlete development. High school baseball players who go undrafted often have to choose between 2 year and 4 year institutions whilst balancing the opportunity for playing time and national exposure. Two year institutions include both junior and community colleges where student athletes can compete for two seasons and/or two academic years. Often, these institutions are used as a stepping stone between high school and a 4 year institution or a professional career. Four year institutions include colleges and universities that offer 4 years of athletic eligibility, where students complete courses to earn bachelor’s degrees. Players drafted out of high school must decide between signing a professional contract or furthering their development at a collegiate program to increase their stock in future drafts. Namely, if they are satisfied with the round they were drafted in and/or the contract, they will sign. Conversely, players who are unsatisfied with their perceived value will decline the contract with the hope that more training will lead to a higher selection in future drafts and thus a more substantial contract. If the athlete chooses to sign the professional contract, they still must traverse the many levels of Minor League Baseball. Although high school aged athletes and their families encounter these challenging decisions every year, research has yet to explore the relationship between specific pathways and later career success. Literature often focuses on the sporting pathway that may develop professional players (Ginsburg et al., 2014) or the capability of professional leagues to successfully identify these players, but rarely does it focus on the relationship between the two. Spurr (2000) and Arnette (2014) attempted to analyze this relationship. The former based success on a player participating in one MLB game and sought to determine the influence that position played, draft round, and highest level of education attained may have on such success. This study largely focused on the ability to identify talent and did not attempt to analyze career success at the MLB level. Arnette (2014) compared the performance of position players (i.e., infielders, outfielders, and catchers) drafted from high school to the performance of players drafted from National Collegiate Athletic Association (NCAA) institutions. Performance was quantified with baseball’s on-base plus slugging (OPS) metric and pitchers were excluded from analyses. Although these studies were important to the talent identification field, they do not assess the significant variability in each pathway and/or include modern performance indicators.

The purpose of this study was to explore the different pathways to MLB and their influence on career success. To achieve a granular description of post-high school pathways, an exploratory approach was used to document all possible pathways to MLB. Once established, we quantitatively assessed the commonality of these pathways in a substantial sample and assessed their effect on career success using modern performance indicators.

## Materials and Methods

### Sample

Our sample consisted of American-born professional baseball players who registered at least one MLB game played between the 1990 and 2010 seasons (*N* = 2, 291). Players were included if their professional debut occurred after April 9th, 1990 (opening day of the 1990 season) and their last professional appearance occurred before November 1st, 2010 (final game of the 2010 season). These dates were selected to establish a 20 year window as research suggests the average career length of an MLB position player is 5.6 years (Witnauer et al., 2007). Thus, a window of 20 years allows the average career player to turn over almost 4 times. Additionally, these specific dates were selected to ensure that all players were truly retired yet still participated in an era fairly representative of modern baseball strategy and evaluation. All players who were not born in the United States of America and/or never registered one game played, were excluded from this sample. For example, American-born players who may have been drafted by a professional baseball team but never registered one official game played in MLB were excluded.

Data were collected from www.baseball-reference.com using player IDs that were collated in a previous dataset (Lemez et al., 2016). Each player ID was entered into Baseball-reference’s search engine allowing compilation of career information and statistics. To ensure the validity of these data, we compared values to additional online sources of baseball information such as www.thebaseballcube.com, www.mlb.com, and www.fangraphs.com. Complete agreement was found amongst each website for draft and career statistics with the exception of Wins Above Replacement (Baseball Prospectus and FanGraphs have their own variations of this metric that differ in the weighting of certain statistics). Pathway information, such as competing at a 2 year or 4 year institution, was verified via the official athletics webpage of the corresponding institution.

### Performance Metrics

Three performance indicators, age of MLB debut, career GP, and Baseball-Reference’s Wins Above Replacement (bWAR) were collected to quantify success. Younger age of debut may be an indication of precocious performance and/or talent (MLB age of debut was computed using date of birth and date of first MLB game). Intuitively, the number of games a player participates in at the professional level is an adequate representation of their career success. This is particularly true for MLB as it drafts the most players and has the most extensive developmental system of the four major North American sports. For example, 32,237 athletes were drafted into MLB between the 1990 and 2010 seasons (Baseball-Almanac, 2019; MyMLBdraft.com, 2019). In the same time frame, 5,490 and 5,345 players were drafted into the NFL and NHL, respectively (Hockey-reference, 2019; Thefootballdb, 2019). This does not include athletes who signed as undrafted free agents, nor does it capture the difficulty of being drafted. Thus, the efficacy of using career GP to indicate career success stems from the immense competition pool traditionally seen in MLB where only the best participate.

To clearly illustrate the value of the athletes to their respective teams, we needed to supplement GP with an additional metric that quantified in-game performance. This was accomplished with bWAR. Simply put, bWAR is www.baseball-reference.com’s adaptation of a measurement that compares the value of a player relative to the value of a replacement level player that may be available (Baseball-reference, 2019b). This measurement is calculated on an interval scale where a positive number demonstrates that a player is contributing to more wins than a replacement level player, hence Wins Above Replacement. For instance, as of the conclusion of the 2018 season, Albert Pujols possesses the highest active bWAR with 100 (Baseball-reference, 2019a). This suggests that Pujols has contributed, in comparison to an average player, 100 more wins to his team throughout his career. While bWAR does not have the traditional appeal that batting average and earned run average provide, it is one of the only statistics that accounts for multifaceted performance (i.e., hitting, defense, pitching, and base running). Furthermore, the possibility of achieving a negative bWAR (i.e., a player contributes less to wins than a replacement level player) ensures that this statistic is not heavily cofounded by the number of GP.

### Pathway

Pre-draft data were used to descriptively delineate the different developmental pathways that athletes in this sample experienced before their MLB debut. Pathways were modeled around transfers between post-secondary institutions (i.e., transferring from a 2 year institution to a 4 year institution) and the decision to sign with a team once drafted (MLB allows you to be drafted multiple times).

Minor League Baseball (MiLB) GP were also collected at three levels to reflect time spent in the MLB’s developmental leagues. This developmental system is traditionally structured so that a player is drafted and/or signed and placed in an “A” league. As a player gains experience and performs, they typically progress to “AA” and “AAA” leagues. Although this is the format of the developmental system, MLB teams may assign a player under contract to any league they choose. Also, a player may be demoted or promoted between leagues at the discretion of the MLB team that has contracted them.

### Analyses

#### Pathways

Descriptive statistics were used to summarize each of the different pathways to MLB. The number of athletes, as well as average (and standard deviation) GP and bWAR within each pathway, were computed. Once descriptive statistics were generated, the data were inspected to determine if there were opportunities to dummy code pathways for the purpose of statistical analyses.

#### Pathway and MLB Performance

All analyses of career performance were performed separately for pitchers and position players because pitchers typically accrue MLB GP at a much slower rate than position players. For instance, Brad Ziegler had the most regular season MLB GP by a pitcher in 2018 with 82. Comparatively, seven position players earned 162 MLB GP in 2018 (i.e., they played in every game of the regular season). One-way MANOVAs were performed to test if the last institution attended influenced athlete’s professional performance or the route to MLB. MLB GP and bWAR, MiLB GP, and MLB debut age were used as dependent variables. The last institution attended (i.e., high school, 2 year institution, or 4 year institution) was the independent variable. To determine the significance of these multivariate tests, Pillai’s criterion (Norman and Streiner, 2008) and Tukey’s Honest Significant Difference (HSD) tests were used for *post hoc* analyses.

#### Pathway and Minor League Baseball Trajectory

Beyond the athlete’s performance at the MLB level, we wanted to determine if the last institution affected the trajectory in MiLB. To do this, another one-way MANOVA was run with “last institution” as the independent factor with A, AA, and AAA GP used as the dependent variables. Pillai’s criterion and Tukey’s HSD were again used to determine significant differences between the pathways. Furthermore, simple linear regressions were calculated to quantify the relationship between MLB GP and bWAR based on the amount of GP at each MiLB level (A, AA, and AAA). Standardized Beta Coefficients are reported to depict these models.

#### Pathway and Variation in MLB Career Performance

To understand what could lead to variation in MLB success, players were grouped depending on their percentile ranking in performance. Frequency analyses were conducted so that position players and pitchers were coded as top 20%, middle 60%, or bottom 20% in MLB GP. A chi-square test of independence was performed to examine the relationship between last institution attended and MLB GP tier (i.e., top, middle, and bottom). Additionally, one-way MANOVAs were conducted for position players and pitchers with MLB GP tier identified as the independent factor and bWAR, MiLB GP, and MLB debut age used at the dependent variables. Significant differences between the dependent variables were determined using Pillai’s criterion and Tukey’s HSD further assessed these differences.

Analyses were performed using SPSS version 25, and statistical significance was defined as *p* < 0.05, at the 95% Confidence Interval (CI).

## Results

### Pathway Grouping

**FIGURE 1:**
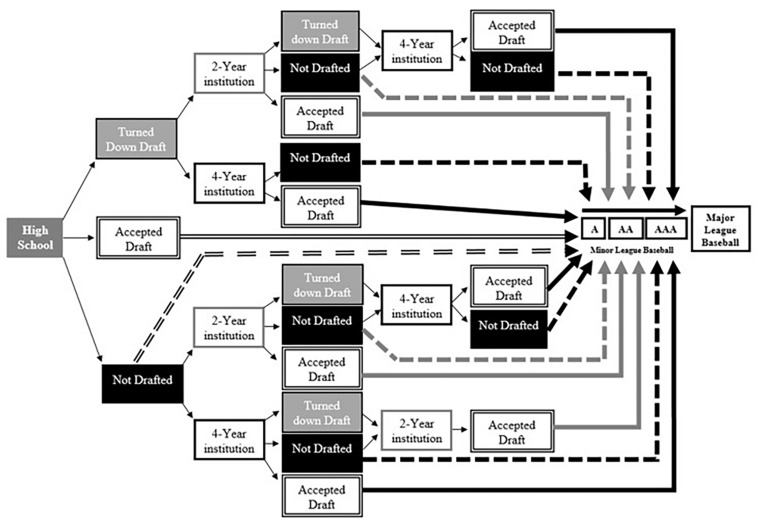
Schematic illustration of the developmental pathways to MLB. Solid arrows represent players signing after being drafted whereas dashed arrows represent players signing as undrafted free agents. Degree of shading and design of arrow pointing to Minor League Baseball represents last institution attended. Single black line = 4 year institution, single gray line = 2 year institution, and double black line = high school. Not Drafted = players were not selected in the draft by a professional team. Turned down Draft = players were selected in the draft by a professional team but elected to turn down the contract associated with their selection. All players are draft eligible after high school graduation, and players at 4 year institutions are draft eligible 3 years after enrolling or after their 21st birthday.

Seventeen descriptively different pathways to MLB were identified (see Figure 1). Pathways were identified post-data collection. The drafting or non-drafting of an athlete from an institution was a key differentiator between pathways, as well as the choice to accept or turn down the draft. Of the 17 different pathways, five represented approximately 83% of MLB players in this sample (see Table 1). To manage the number of different groups for statistical analyses, pathways were then categorized into one of three main streams. These categories were determined by last institution attended and identified as high school, 2 year institution, and 4 year institution. Namely, players were sorted by the last institution attended directly before signing a professional MLB contract. This was selected as a common theme as every player in the sample began their professional baseball career after attending at least one of these three main institutions.

**TABLE 1:** General pathways and their MLB performance.

			**MLB**	
			
**Pathway**	**Description**	**Athletes**	**GP**	**bWAR**
		**(N)**	***M* (*SD*)**	***M* (*SD*)**
1	High school draft → 4 year draft	334	233.8 (385.3)	2.5 (8.3)
2	High school draft	512	261.4 (418.4)	2.5 (7.2)
3	2 year draft	189	198.1 (341.7)	1.9 (6)
4	4 year draft	740	211.9 (363)	2.1 (7.2)
5	2 year draft → 4 year draft	116	194.7 (301.8)	1.6 (4.7)
6	High school draft → 4 year NO draft	14	320.2 (627.9)	2.1 (5.3)
7	2 year draft → 4 year NO draft	19	199.7 (348.9)	1.3 (3.6)
8	High school draft → 2 year draft	60	223.4 (391.1)	2.1 (6)
9	2 year NO draft → 4 year draft	102	172.5 (263.5)	2.1 (5.9)
10	High school draft → 2 year draft → 4 year draft	42	139.5 (247.2)	1.1 (4.5)
11	4 year draft → 2 Year draft	9	341.7 (246.2)	6.6 (5.4)
12	NO draft 4 year	58	132.9 (292.6)	1 (3.9)
13	NO draft 2 year	15	161.9 (221.1)	1.6 (5.5)
14	NO draft high school	21	155.9 (156.5)	0.7 (3.1)
15	High school draft → 2 year NO draft	30	161.2 (224.5)	1.4 (3.6)
16	4 year NO draft → 2 year draft	4	811.8 (871)	17.2 (28.4)
17	2 year NO draft → NO 4 year draft	26	179.7 (330.2)	2.2 (4.6)
	Total	2291	219.6 (368.9)	2.2 (7)

*High school, high school institution; 2 year, 2 year institution; 4 year, 4 year institution; MLB, Major League Baseball; GP, Games Played; bWAR, Baseball Reference’s Wins Above Replacement. → Indicates a transfer between institutions. Pathways that end in NO Draft suggest that the player signed as an undrafted free agent; M, mean; SD, standard deviation.*

### Pathway and MLB Performance

#### Position Players

A significant difference in MLB debut age (*p* < 0.001, η*_*p*_*^2^ = 0.13) and the amount of MiLB GP (*p* < 0.001, η*_*p*_*^2^ = 0.04) based on last institution attended, *F*(8, 1,480) = 28.61, *p* < 0.001; V = 0.27, η*_*p*_*^2^ = 0.13 (see Table 2) was observed. No significant differences between MLB GP and bWAR were noted. *Post hoc* analyses suggested that high school position players debuted younger and played in more MiLB games than position players from 2 year and 4 year institutions (see Table 2).

**TABLE 2:** MLB and MiLB descriptive statistics – position players.

**Last institution attended**	**Athletes (N)**	**MLB GP *M* (*SD*)**	**bWAR *M* (*SD*)**	**MiLB GP *M* (*SD*)**	**MLB debut age *M* (*SD*)**
High school	170	326.82 (483.55)	2.44 (6.71)	909.13^∗∗^ (350.83)	23.99^∗∗^ (2.11)
2 year	87	304.79 (412.61)	2.09 (6.26)	834.41 (292.35)	25.67 (2.02)
4 year	488	278.36 (408.38)	1.85 (5.56)	752.33 (307.2)	25.95 (2.24)

*MLB, Major League Baseball; GP, Games Played; bWAR, Baseball Reference’s Wins Above Replacement; MiLB, Minor League Baseball; M, mean; SD, standard deviation; N, number; ^∗∗^p < 0.01.*

#### Pitchers

Pitchers also demonstrated a significant difference in MLB debut age (*p* < 0.001, η*_*p*_*^2^ = 0.13) and MiLB GP (*p* < 0.01, η*_*p*_*^2^ = 0.01) as well as in MLB GP (*p* < 0.05, η*_*p*_*^2^ = 0.01) based on last institution attended, *F*(8, 3,082) = 31.96, *p* < 0.001; V = 0.15, η*_*p*_*^2^ = 0.08 (see Table 3). *Post hoc* analyses revealed the same trend for pitchers as was seen in position players (younger MLB debut and more MiLB GP) but also showed that high school pitchers averaged more MLB GP than pitchers from a 4 year institution (see Table 3).

**TABLE 3:** MLB and MiLB descriptive statistics – pitchers.

**Last institution Attended**	**Athletes (N)**	**MLB GP *M* (*SD*)**	**bWAR *M* (*SD*)**	**MiLB GP *M* (*SD*)**	**MLB debut age *M* (*SD*)**
High school	363	224.67^∗^ (369.53)	2.47 (7.32)	366.43^∗∗^ (289.07)	23.80^∗∗∗^ (1.90)
2 year	220	172.32 (316.24)	2.23 (6.80)	331.41 (240.69)	25.10 (2.09)
4 year	963	172.10 (319.07)	2.21 (7.56)	316.15 (257.30)	25.62 (1.91)

*MLB, Major League Baseball; GP, Games Played; bWAR, Baseball Reference’s Wins Above Replacement; MiLB, Minor League Baseball; M, mean; SD, standard deviation; N, number; ^∗^p < 0.05, ^∗∗^p < 0.01, ^∗∗∗^p < 0.001.*

### Pathway and Minor League Baseball Trajectory

#### Position Players

**FIGURE 2:**
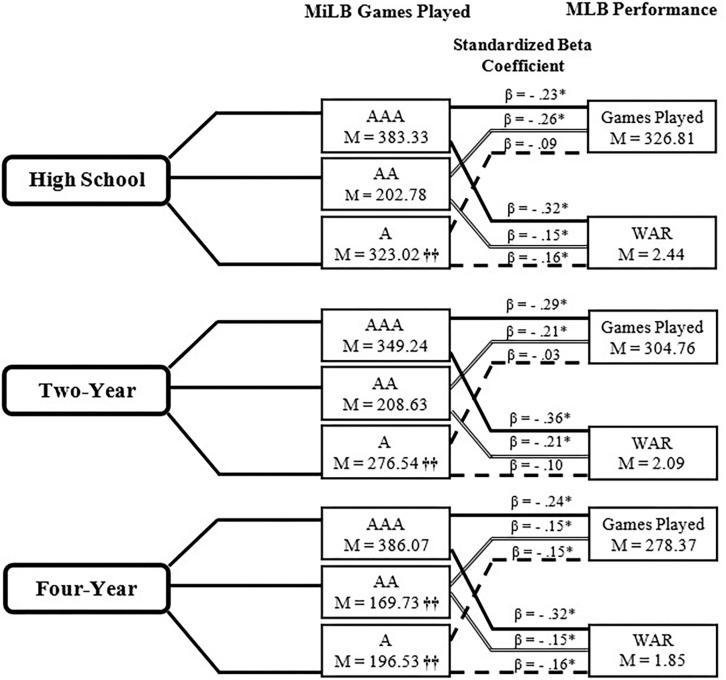
Relationship between Minor League career and MLB performance- Position Players. WAR, Wins Above Replacement. ^†⁣†^Significant difference (*p* < 0.05) from two other institutions at that level. β Signifies standardized beta coefficient from regression model with A, AA, AAA Games Played used as predictors of Major League Baseball Games Played and Wins Above Replacement; ^∗^*p* < 0.05.

Position players demonstrated notably different MiLB trajectories based on the last institution attended. Specifically, “AA” (*p* < 0.001, η*_*p*_*^2^ = 0.02) and “A” (*p* < 0.001, η*_*p*_*^2^ = 0.20) GP differed significantly as a function of last institution attended *F*(6, 1,482) = 29.86, *p* < 0.001; V = 0.22, η*_*p*_*^2^ = 0.11 (see Figure 2). High school position players played significantly more “A” games than both 2 year and 4 year players. Additionally, they played significantly more “AA” and total MiLB games than players who were selected or signed after attending a 4 year institution (see Figure 2). No significant differences were noted at the “AAA” level.

Regression analyses revealed a statistically significant negative relationship between number of GP at AAA and AA MiLB and subsequent MLB GP for high school, 2 year, and 4 year pathway athletes. In addition, there was a statistically significant negative relationship between GP at A MiLB and MLB GP for 4 year pathway athletes (see Figure 2). The same results were found for the 2 year pathway, however, the relationship between GP at A MiLB and MLB GP was not statistically significant (Figure 2). Overall, more GP in MiLB is related to fewer MLB GP. The analyses between GP at AAA, AA and A MiLB and subsequent MLB WAR also demonstrated a negative relationship. With the exception of A-level GP for the 2 year pathway, this negative relationship was statistically significant for each pathway and each level of MiLB. The magnitude of this relationship was largest for AAA MiLB GP and MLB GP, and similar in size for each pathway (β range: −0.32 to −0.36). Like GP, the more GP in MiLB the lower WAR score in MLB.

#### Pitchers

**FIGURE 3:**
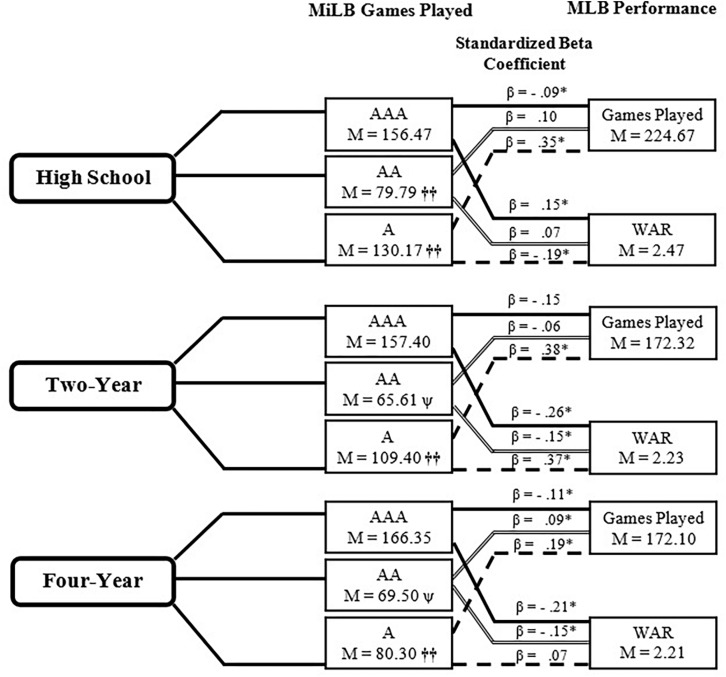
Relationship between Minor League career and MLB performance- Pitchers. WAR, Wins Above Replacement, ^†⁣†^Significant difference (*p* < 0.05) from two other institutions at that level. ΨSignificant difference (*p* < 0.05) from High School institution at that level. β Signifies standardized beta coefficient from regression model with A, AA, AAA Games Played used as predictors of Major League Baseball Games Played and Wins Above Replacement; ^∗^*p* < 0.05.

Pitchers participated in significantly different AA (*p* = 0.01, η*_*p*_*^2^ = 0.01), A (*p* < 0.001, η*_*p*_*^2^ = 0.06) and total MiLB GP (*p* < 0.01, η*_*p*_*^2^ = 0.01) based on last institution attended *F*(6, 3,084) = 26.85, *p* < 0.001; V = 0.10, η*_*p*_*^2^ = 0.05 (see Figure 3). High school pitchers played significantly more A and AA games than pitchers from a 2 year institution and significantly more A, AA, and total MiLB games than 4 year pitchers. No significant differences were noted at the AAA level.

There was a consistent, and statistically significant, *positive* relationship between GP at A MiLB and MLB GP for each of the three pathways. The magnitude of this relationship was largest for high-school (0.35) and 2 year (0.38) pathways. In contrast, there was a negative relationship between GP at AAA MiLB and MLB GP (only statistically significant for the high-school and 4 year pathways). With respect to MLB WAR, there were no consistent patterns. For those emerging directly from high-school, there was a positive relationship between GP at AAA and MLB WAR, but a negative relationship between GP at A and MLB WAR (both statistically significant). For athletes emerging from the 2 year pathway, there were statistically significant negative relationships between GP at AAA and AA MiLB, and MLB WAR, while there was a positive relationship between GP at A MiLB and MLB WAR (statistically significant). The 4 year pathway demonstrated the same relationship as the 2 year pathway, although only the AAA and AA GP demonstrated statistically significant relationships with MLB WAR.

### Pathway and Variation in MLB Career Performance

#### Position Players

**FIGURE 4:**
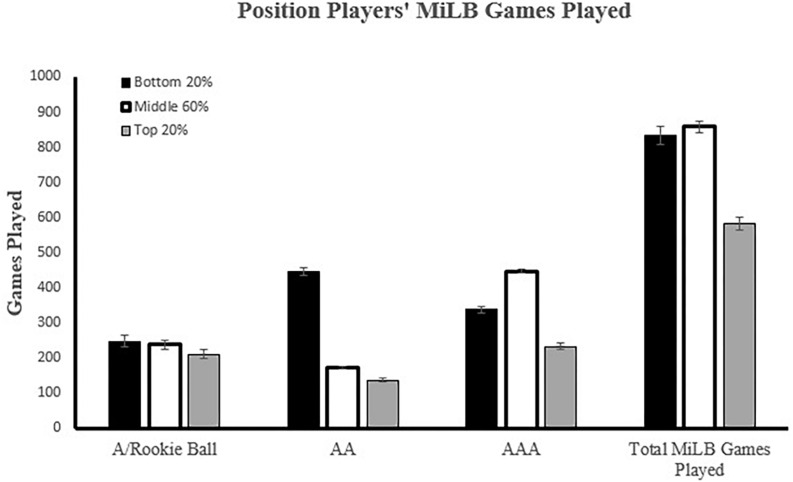
Position Players’ relationship between Minor League Baseball Games Played (MiLBGP) and Major League Baseball Games Played (MLBGP) performance. MLB GP sorted into percentiles: **Top** 20%, **Middle** 60%, **Bottom** 20%.

Chi-square results of position players suggested that last institution attended was evenly distributed across each tier of MLB GP, χ^2^(4, *N* = 745) = 3.88, *p* = 0.42, φ_c_ = 0.05. However, significant differences in bWAR (*p* < 0.001, η*_*p*_*^2^ = 0.31), MiLB GP (*p* < 0.001, η*_*p*_*^2^ = 0.08) and MLB debut age (*p* < 0.001, η*_*p*_*^2^ = 0.08) when grouped by tier of MLB GP, *F*(6, 1,470) = 53.56, *p* < 0.001; V = 0.36, η*_*p*_*^2^ = 0.18 (see Figure 4) were noted. Position players who finished in the top 20% of MLB GP averaged a higher bWAR, played significantly fewer MiLB GP, and debuted in the MLB at a younger age than players that finished in the middle 60% and bottom 20%. Additional analyses revealed that position players who finished in the top 20% of MLB GP played significantly fewer games at every minor league level, *F*(6, 1,482) = 34.58, *p* < 0.001; V = 0.25, η*_*p*_*^2^ = 0.12 (see Figure 4).

#### Pitchers

**FIGURE 5:**
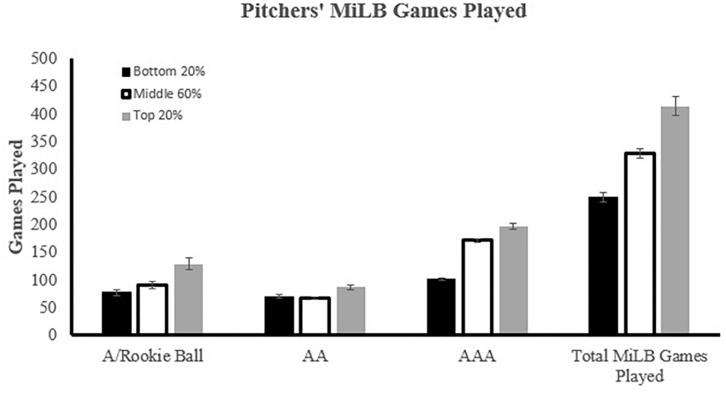
Pitchers’ relationship between Minor League Baseball Games Played (MiLBGP) and Major League Baseball Games Played (MLBGP) performance. MLBGP sorted into percentiles: **Top** 20%, **Middle** 60%, **Bottom** 20%.

Unlike position players, chi-square results of pitchers suggested that last institution attended was not evenly distributed across each tier of MLB GP, χ^2^(4, *N* = 1546) = 10.82, *p* < 0.05, φ_c_ = 0.06. The asymmetry in distribution stems from the underrepresentation of 2 year players and overrepresentation of 4 year players, relative to expected counts, in the bottom 20% of MLB GP. Although this value was statistically significant, the low Cramer’s V suggests that practical significance is lacking. Pitchers also demonstrated significant differences in bWAR (*p* < 0.001, η*_*p*_*^2^ = 0.21), MiLB GP (*p* < 0.001, η*_*p*_*^2^ = 0.03), and MLB debut age (*p* < 0.001, η*_*p*_*^2^ = 0.04) when grouped by tier of MLB GP, *F*(6, 3,072) = 88.04, *p* < 0.001; V = 0.29, η*_*p*_*^2^ = 0.15 (see Figure 5). Similar to position players, pitchers who finished in the top 20% of MLB GP averaged a higher bWAR and debuted in the MLB at a younger age when compared to the middle 60% and bottom 20%. However, pitchers were different from position players in that the top 20% averaged significantly more MiLB GP. In fact, pitchers who finished in the top 20% of MLB GP played significantly *more* games at every minor league level, *F*(6, 3,084) = 21.02, *p* < 0.001; V = 0.08, η*_*p*_*^2^ = 0.04 (see Figure 5).

## Discussion

The purpose of this study was to explore the variation in pathways to MLB, and assess their influence on career performance. We observed 17 independent pathways, spanning from being drafted out of high-school to attending a 4 year college, then participating in MiLB, before ultimately reaching MLB (with varying degrees of success). This variation is certainly consistent with the notion that pathways to elite levels of performance are rarely straightforward or linear (Gulbin J. et al., 2013). However, the current results also stress the need to consider how variations in *the contexts in which* athletes participate during the same, or similar, stages can have ambiguous influences on athlete development. The 17 pathways were sorted into three main categories based on last institution attended before entering professional baseball. Overall, there were asymmetries in the proportion of athletes from distinct athlete development pathways. Consistent with past research (Spurr, 2000), more players were drafted out of college; approximately 77% of players in the sample attended a post-secondary institution and were directly drafted or signed from there.

With respect to the different pathways, high-school-drafted pitchers and position players played significantly more games in MiLB, particularly at the A level. This suggests that A-level MiLB may be somewhat equivalent to college/university baseball with respect to competition and training quality. Alternatively, the characteristics of the athletes at this stage in their development (e.g., growth, maturation, skill, and/or ability) may align favorably with the characteristics of both MiLB-A and college/university (Hoffman et al., 2009). Interestingly, no significant differences between the three pathways were noted in the amount of GP at the “AAA” level. This may be indicative of a “wash-out” effect of last institution attended in the minor leagues. Once the players reach the AAA level, it seems like the past baseball experiences of these players level off. However, the finding that high-school-drafted athletes were found to debut in MLB at significantly younger ages than athletes from the college/university pathways suggests that the trade-off, or equivalency, might not be exact. This may be due to several factors. Athletes drafted out of high-school may be viewed as precocious, or having exceptional potential (accurately or inaccurately), resulting in their promotion through MiLB at a faster rate than typically seen through the college/university pathway. Athletes who choose to develop in the college/university pathways (particularly the 4 year route) may simply be inclined to finish their degrees and/or may have greater commitment to their teams (e.g., greater group and task cohesion; see Eys et al., 2015). It is also possible that high-school-drafted athletes start their minor league careers younger (typically 18 years old), allowing them to accrue the necessary A and AA games needed to assess their ability at a younger age than college/university players.

While the MLB debut ages were different amongst the three pathways, this did not appear to influence overall career success. Although the high-school-pathway pitchers, with younger debut ages, had a greater number of total MLB GP, there were no significant differences between the pathways with respect to WAR. The lack of a relationship between GP and WAR for the high-school-pathway pitchers could support the existence of a sunk cost effect (see Koz et al., 2012; Keefer, 2018). Perhaps their identification as precocious, and worthy of being drafted out of high-school, predisposes coaches and administrators to invest time and opportunities for these athletes to play despite evidence to the contrary. Future research should address this by exploring age of debut as a verifiable performance indicator, particularly for pathways that may be subject to the sunk cost effect. The fact that the three main developmental pathways are not directly related to career success is also supported by their equal representation in the top 20% of GP at MLB. Although these pathways may contain ostensible differences, it is possible that the developmental experiences accumulated within each pathway mediate and/or moderate later career success. Further inquiries into this concept may have fruitful implications for pathway and talent identification research.

While there was no direct relationship between the three primary developmental pathways and career success, results did suggest there are indicators at the MiLB level of future MLB career success, and that these indicators differ according to playing position. The negative relationships between AAA and AA GP and MLB GP *and* WAR in position players suggest more GP at these levels is associated with decreased MLB career success. This finding appears to indicate that less-skilled players are spending more time at MiLB levels and are unable to successfully transition to the MLB. The 4 year cohort also displayed a significant, negative relationship between “A” GP and MLB GP/WAR, likely because they are not expected to spend as much time at this level (because of their college experience). As such, players who play more games at the A level are presumably not as talented as those that quickly progress past this level.

Similar trends were observed for *pitchers*, with a negative relationship between AAA GP and MLB GP and WAR. However, a significant positive correlation was observed between A GP and MLB GP, along with finding that pitchers in the top 20% of MLB GP played more total MiLB games than pitchers in the bottom 80% of GP. These results are opposite to what was found for position players, and this discrepancy is difficult to account for. Perhaps young talented pitchers must flourish at A with more GP before progressing to higher levels. Spurr and Barber (1994) explored the relationship between total time spent at A and the outcome for a pitcher (promotion or demotion). Results suggested the amount of time spent at this level was related to performance metrics (i.e., Earned Run Average and strikeouts), and the more a pitcher diverged from the league mean in either direction, the quicker a decision was made about their outcome. These results, in accordance with the positive correlation observed between A GP and MLB GP, suggests that pitchers who demonstrate consistent performance (close to or above league average) may be afforded more opportunities in the MLB. There may also be unique task constraints to pitching that require additional developmental time (i.e., MiLB GP) to acquire strength and refine skill acquisition and consistency. Indeed, an abundance of research has outlined the biomechanical intricacies involved in elite pitching (Stodden et al., 2001, 2005) and possible mechanisms that facilitate acquisition of skilled throwing (Fleisig et al., 1999; Stodden et al., 2006). It is also possible that the more games you appear in as a pitcher, the better you are/more chances you will get at the MLB level. Future research would benefit from exploring the potential mechanisms that contribute to the different developmental patterns of pitchers and position players. For example, it may be useful to include performance metrics at MiLB (e.g., earned run average for pitchers and batting average for position players) in future studies on MLB athlete development.

Regarding stage based development models (i.e., DMSP, FTEM, and LTAD), the present study illuminates the notable variation that may be present within different stages, particularly past adolescents. Although these models attempt to maximize generalizability, it is important that nuances in athlete development are not overlooked. Future research and models should strive to address diverse pathways and to understand what qualities make a specific pathway optimal for certain athletes. These findings may also be of interest to young players and their families when they are deciding which pathway to embark on (i.e., when balancing the pros and cons). However, additional research is needed to understand why athletes choose certain pathways. Factors such as their personal characteristics, socioeconomic status, aptitude for higher education, and geographic location of development likely influence this decision in variable ways. As such, it is important to note that this study was exploratory in nature and serves as a starting point for understanding non-linear development. Indeed, more comprehensive work in this area could lead to better identification of desirable developmental experiences, particularly within the developmental trajectories to MLB.

While this study provided some novel information about athlete development, it was not without limitations. We did not include athletes who were drafted but did not play a game at the MLB level. Not including this information may skew our understanding of the effectiveness of each pathway in terms of athlete development. It may be interesting to explore if one of the pathways, or MiLB indicators, is disproportionately related to the likelihood of never reaching MLB. Furthermore, while we were interested in exploring the variations in athlete development associated with different pathways, we collapsed the 17 independent pathways down to three for the purposes of analyses. While this decision was based on sample size and commonalities between similar pathways, there may have been interesting variations in athlete development *within* the three pathway groups. When interpreting our results, it is also necessary to acknowledge the debate between statistics, such as WAR. We chose to use bWAR as an indicator of MLB career success for its ease of accessibility and comprehension. However, others advocate the use of alternative WAR metrics (see Baumer et al., 2015, for a review of WAR statistics and the advocacy for openWAR). While we do not believe that a different WAR metric would dramatically alter our conclusion, it is possible that it could alter some of our results.

In summary, the heterogeneity of athlete development pathways in this and other sports is becoming increasingly relevant for practitioners working in sport. The findings in this study are indicative of non-linear development, and demonstrate that the pathways to success are diverse. Furthermore, operationalization of the term success in athlete development and talent identification literature appears to be subjective. As such, identification of desirable developmental pathways and optimal characteristics within each pathway may be contingent on how success is contextualized. Specific to MLB, while career success can be defined from several different vantage points, acknowledging the developmental variance leading to elite-performance involvement is important for a more complete understanding of the challenges faced by talent identification and development systems.

## Data Availability Statement

All datasets collated for this study were generated from publicly available information. The raw data supporting the conclusions of this manuscript will be made available by the authors, without undue reservation, to any qualified researcher.

## Ethics Statement

This study was exempt from ethical approval procedures as all data is publicly available and retrospective.

## Author Contributions

MM and NW contributed to the conception and design of the study, and performed the statistical analysis. MM organized the database and wrote the first draft of the manuscript. All authors wrote sections of the manuscript, contributed to manuscript revision, and read and approved the submitted version.

## Conflict of Interest

The authors declare that the research was conducted in the absence of any commercial or financial relationships that could be construed as a potential conflict of interest.

## References

[B1] Arnette G. III (2014). *The Path to the Top: How Career Paths Differ Between College and Non-College Major League Baseball Players.* Clemson: Clemson University.

[B2] Baseball-Almanac (2019). *Baseball Draft History.* Available at: http://www.baseball-almanac.com/draft/baseball_draft.shtml (accessed February 9, 2019).

[B3] Baseball-reference (2019a). *Active Leaders & Records for Wins Above Replacement.* Available at: https://www.baseball-reference.com/leaders/WAR_active.shtml (accessed February 9, 2019).

[B4] Baseball-reference (2019b). *WAR Explained.* Available at: https://www.baseball-reference.com/about/war_explained.shtml (accessed February 9, 2019).

[B5] Baumer B. S. Jensen S. T. Matthews G. J. (2015). openWAR: an open source system for evaluating overall player performance in major league baseball. *J. Q. Anal. Sports* 11 69–84.

[B6] Beneventano P. Berger P. D. Weinberg B. D. (2012). Predicting run production and run prevention in baseball: the impact of Sabermetrics. *Int. J. Bus. Humanit Technol.* 2 67–75.

[B7] Costa G. B. Huber M. R. Saccoman J. T. (2014). *Understanding Sabermetrics: an Introduction to the Science of Baseball Statistics.* Jefferson, NC: McFarland.

[B8] Côté J. Baker J. Abernethy B. (2007). Practice and play in the development of sport expertise. *Handb. Sport Psychol.* 3 184–202. 10.1002/9781118270011.ch8

[B9] Eys M. Loughead T. M. Godfrey M. (2015). “Group cohesion and athlete development,” in *Routledge Handbook of Talent Identification and Development in Sport*, eds Baker J. Cobley S. Schorer J. Wattie N. (London: Routledge), 301–311. 10.4324/9781315668017-21

[B10] Fleisig G. S. Barrentine S. W. Zheng N. Escamilla R. F. Andrews J. R. (1999). Kinematic and kinetic comparison of baseball pitching among various levels of development. *J. Biomech.* 32 1371–1375. 10.1016/s0021-9290(99)00127-x 10569718

[B11] Fraser-Thomas J. Côté J. (2009). Understanding adolescents’ positive and negative developmental experiences in sport. *Sport Psychol.* 23 3–23. 10.1123/tsp.23.1.3

[B12] Ginsburg R. D. Smith S. R. Danforth N. Ceranoglu T. A. Durant S. A. Kamin H. (2014). Patterns of specialization in professional baseball players. *J. Clin. Sport Psychol.* 8 261–275. 10.1123/jcsp.2014-0032

[B13] Gulbin J. Weissensteiner J. Oldenziel K. Gagné F. (2013). Patterns of performance development in elite athletes. *Eur. J. Sport Sci.* 13 605–614. 10.1080/17461391.2012.756542 24251738

[B14] Gulbin J. P. Croser M. J. Morley E. J. Weissensteiner J. R. (2013). An integrated framework for the optimisation of sport and athlete development: a practitioner approach. *J. Sports Sci.* 31 1319–1331. 10.1080/02640414.2013.781661 23631711

[B15] Hockey-reference (2019). *NHL Entry and Amateur Draft History.* Available at: https://www.hockey-reference.com/draft/ (accessed February 9, 2019).

[B16] Hoffman J. R. Vazquez J. Pichardo N. Tenenbaum G. (2009). Anthropometric and performance comparisons in professional baseball players. *J. Strength Condit. Res.* 23 2173–2178. 10.1519/JSC.0b013e3181bcd5fe 19826310

[B17] Keefer Q. A. W. (2018). Decision-maker beliefs and the sunk-cost fallacy: major league baseball’s final-offer salary arbitration and utilization. *J. Econom. Psychol.* (in press). 10.1016/j.joep.2018.06.002

[B18] Koz D. Fraser-Thomas J. Baker J. (2012). Accuracy of professional sports drafts in predicting career potential. *Scand. J. Med. Sci. Sports* 22 e64–e69. 10.1111/j.1600-0838.2011.01408.x 22092367

[B19] Lemez S. Wattie N. Baker J. (2016). Early death in active professional athletes: trends and causes. *Scand. J. Med. Sci. Sports* 26 544–549. 10.1111/sms.12480 25996659

[B20] Massey C. Thaler R. H. (2013). The loser’s curse: decision making and market efficiency in the national football league draft. *Manag. Sci.* 59 1479–1495. 10.1287/mnsc.1120.1657

[B21] MyMLBdraft.com (2019). *MLB Draft Results.* Available at: http://www.mymlbdraft.com/2010/round1/ (accessed February 9, 2019).

[B22] Norman G. R. Streiner D. L. (2008). *Biostatistics: the Bare Essentials.* New York, NY: BC Decker Inc.

[B23] Popper S. (2004). Draft strategy: how has it changed. *Athlon Sports Pro Basketball* 11 19–21.

[B24] Simpson A. G. (1990). *The Baseball Draft: The First 25 Years, 1965-1989.* Sinking Spring, PA: American Sports Pub.

[B25] Spurr S. J. (2000). The baseball draft: a study of the ability to find talent. *J. Sports Econom.* 1 66–85. 10.1177/152700250000100106

[B26] Spurr S. J. Barber W. (1994). The effect of performance on a worker’s career: evidence from minor league baseball. *ILR Rev.* 47 692–708. 10.1177/001979399404700412

[B27] Staw B. M. Hoang H. (1995). Sunk costs in the NBA: why draft order affects playing time and survival in professional basketball. *Admin. Sci. Q.* 40 474–494.

[B28] Stodden D. F. Fleisig G. S. McLean S. P. Andrews J. R. (2005). Relationship of biomechanical factors to baseball pitching velocity: within pitcher variation. *J. Appl. Biomech.* 21 44–56. 10.1123/jab.21.1.44 16131704

[B29] Stodden D. F. Fleisig G. S. McLean S. P. Lyman S. L. Andrews J. R. (2001). Relationship of pelvis and upper torso kinematics to pitched baseball velocity. *J. Appl. Biomech.* 17 164–172. 10.1123/jab.17.2.164

[B30] Stodden D. F. Langendorfer S. J. Fleisig G. S. Andrews J. R. (2006). Kinematic constraints associated with the acquisition of overarm throwing Part I: step and trunk actions. *Res. Q. Exerc. Sport* 77 417–427. 10.5641/027013606x13080770015120 17243217

[B31] Thefootballdb (2019). *NFL Draft History.* Available at: https://www.footballdb.com/draft/index.html (accessed February 9, 2019).

[B32] Weissensteiner J. R. (2015). “Method in the madness: working toward a viable ‘paradigm’ for better understanding and supporting the athlete pathway 133-149,” in *Routledge Handbook of Talent Identification and Development in Sport*, eds Baker J. Cobley S. Schorer J. Wattie N. (London: Routledge).

[B33] Witnauer W. D. Rogers R. G. Saint Onge J. M. (2007). Major league baseball career length in the 20th century. *Populat. Res. Pol. Rev.* 26 371–386. 10.1007/s11113-007-9038-5 21976782 PMC3184466

